# Robust Prognostic Subtyping of Muscle-Invasive Bladder Cancer Revealed by Deep Learning-Based Multi-Omics Data Integration

**DOI:** 10.3389/fonc.2021.689626

**Published:** 2021-08-06

**Authors:** Xiaolong Zhang, Jiayin Wang, Jiabin Lu, Lili Su, Changxi Wang, Yuhua Huang, Xuanping Zhang, Xiaoyan Zhu

**Affiliations:** ^1^School of Electronic and Information Engineering, Xi’an Jiaotong University, Xi’an, China; ^2^Sun Yat-sen University Cancer Center, State Key Laboratory of Oncology in South China, Collaborative Innovation Center for Cancer Medicine, Guangzhou, China; ^3^School of Medicine, Shenzhen University, Shenzhen, China; ^4^Department of Pathology, Sun Yat-sen University Cancer Center, Guangzhou, China

**Keywords:** muscle-invasive bladder cancer, multi-omics, deep learning, subtyping, prognosis

## Abstract

Muscle-invasive bladder cancer (MIBC) is the most common urinary system carcinoma associated with poor outcomes. It is necessary to develop a robust classification system for prognostic prediction of MIBC. Recently, increasing omics data at different levels of MIBC were produced, but few integration methods were used to classify MIBC that reflects the patient’s prognosis. In this study, we constructed an autoencoder based deep learning framework to integrate multi-omics data of MIBC and clustered samples into two different subgroups with significant overall survival difference (*P* = 8.11 × 10^-5^). As an independent prognostic factor relative to clinical information, these two subtypes have some significant genomic differences. Remarkably, the subtype of poor prognosis had significant higher frequency of chromosome 3p deletion. Immune decomposition analysis results showed that these two MIBC subtypes had different immune components including macrophages M1, resting NK cells, regulatory T cells, plasma cells, and naïve B cells. Hallmark gene set enrichment analysis was performed to investigate the functional character difference between these two MIBC subtypes, which revealed that activated IL-6/JAK/STAT3 signaling, interferon-alpha response, reactive oxygen species pathway, and unfolded protein response were significantly enriched in upregulated genes of high-risk subtype. We constructed MIBC subtyping models based on multi-omics data and single omics data, respectively, and internal and external validation datasets showed the robustness of the prediction model as well as its ability of prognosis (*P* < 0.05 in all datasets). Finally, through bioinformatics analysis and immunohistochemistry experiments, we found that KRT7 can be used as a biomarker reflecting MIBC risk.

## Introduction

Bladder urothelial carcinoma (BLCA) is one of the most common cancer types in human (1), while muscle-invasive bladder cancer (MIBC) accounts for the majority of patient mortality (2). Over the past tens of years, there is no practical option to improve the survival of MIBC patients. Unlike the high 5-year survival rate (95%) of bladder cancer that has not spread beyond the inner layer of the bladder wall, the 5-year survival rate of MIBC without distant metastasis dropped to 69%, and if cancer extends through the bladder to the surrounding tissue or has spread to nearby lymph nodes or organs, the 5-year survival rate is 35% (Approved by the Cancer.Net Editorial Board, 05/2019).

In recent years, many studies have characterized the molecular features at different omics levels and reported subclassification of bladder cancer into distinct subtypes based on unique molecular signatures (3–11). For example, The Cancer Genome Atlas (TCGA) consortium reported four clusters of MIBCs with gene expression profiling and two of which were also evident in microRNA (miRNA) sequencing and protein data (6). Robertson et al. (11) recruited many TCGA-MIBC samples and subtyped the MIBC patients referring to the mutation signature, the expression of mRNA, lncRNA, and miRNA, respectively, and revealed some of the subtypes related to a poor-survival phenotype.

Nevertheless, the previous studies investigated the molecular subtypes of bladder cancer only based on single omics level, and did not connect with the survival information during the process of defining subtypes. Thus, a subtyping method that could reflect different survival profiles is valuable for the clinical application in guiding the treatment of MIBC patients.

**Figure 1:**
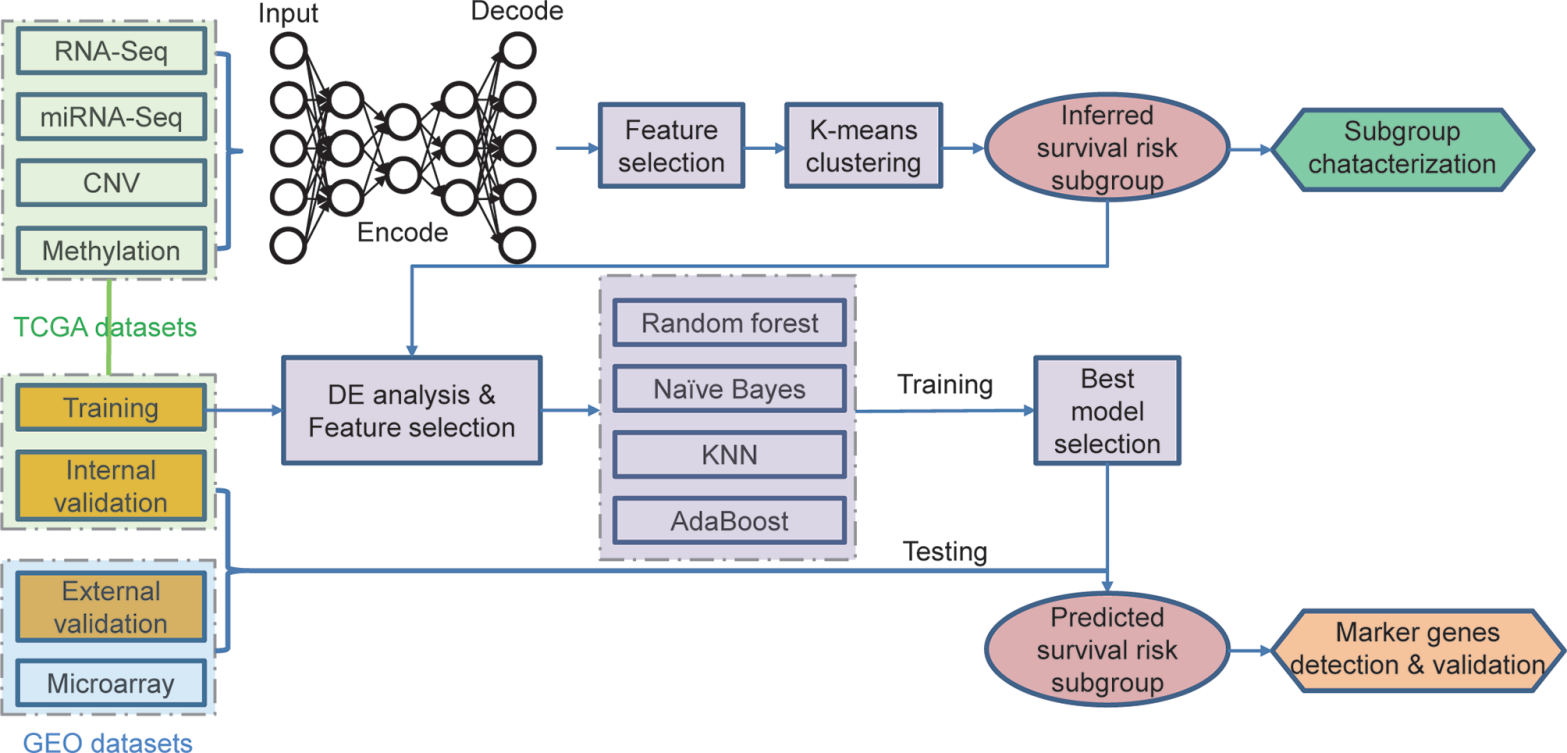
Overall workflow for the deep learning-based prognostic subtyping and validation.

Here, we employed a multi-omics-based utilized deep learning (DL) computational framework to stratify the MIBC patients into two subgroups concerning different risks of overall survival (OS) (**Figure 1**). We investigated feature differences between the two subgroups of MIBC, and derived prognostic models based on multi- or single-omics data to classify MIBC into different subgroups. Gene expression-based model were further validated by both in-group and out-group datasets. Besides, we figure out a cell surface marker—KRT7 (CK7), which is significantly differently expressed in high-risk and low-risk MIBC.

## Materials and Methods

### Datasets and Study Design

The multi-omics data of TCGA-BLCA, including gene-level copy number variation (CNV) profile, mRNA and miRNA expression profile revealed by RNA-seq and miRNA-seq, and DNA methylation data profiled by Illumina Infinium HumanMethylation450 platform, were downloaded from the University of California Santa Cruz (UCSC) Xena database (https://xenabrowser.net/).

Only samples with tumor stage II/III/IV (MIBC) remained for downstream analysis. These TCGA-MIBC datasets were used in two ways: 1) All samples were used to perform subgroup stratification based on deep learning and clustering algorithm; 2) samples were randomly split by 4:1, including a training dataset to train the classification model and an in-group testing dataset to validate the prediction accuracy. Three gene microarray matrices containing 43 MIBC patients (GSE19915), 62 MIBC patients (GSE48277-GPL14951), and 73 MIBC patients (GSE48277-GPL6947) were downloaded from Gene Expression Omnibus (GEO) database (https://www.ncbi.nlm.nih.gov/geo/), serving as out-group validation datasets. For these datasets, only samples with prognostic information were taken into consideration for downstream analysis.

### Multi-Omics Data Integration

The autoencoder framework was chosen as the implementation of deep learning for integrating the results derived from multi-omics data. The CNV, gene expression, miRNA expression, and methylation data extracted from TCGA-MIBC dataset served as an input for the autoencoders framework. The autoencoder was a dimensionality reduction method based on an unsupervised feed-forward, non-recurrent neural network, which is implemented in python with package Keras (https://github.com/fchollet/keras).

We build the autoencoder framework as previously reported (12), which could be briefly described as follows:

For a given input layer, the objective of an autoencoder reconstructed the input layer *x* (sized as *d* × *p*) into the same dimension output layer *y* through an activation function *tanh* (a hidden layer between *x* and *y*). In this study, we used the four preprocessed data matrices of different level of omics data (features × samples) and stacked all features together into a merged big matrix. In total, 350,631 features were used for downstream analysis. All of the features except CNV features were scaled so that all values are within a similar distribution range. This step could be expressed as:

$$y_{i}=f_{i}(x)=tanh(W_{i\cdot }x+b_{i})$$

where *b_i_* is an intercept vector of size p and *W_i·_x = Σ_j_ W_i,j·_x_j_*, in which *x_j_* is the value of a single feature of *x*. When the autoencoder framework has *k* layers,

$$y=F_{1\to k}(x)=f_{1}{}^{\circ}\cdots f_{k-1}{}^{\circ}f_{k}(x)$$

where $f_{k-1}{}^{\circ}f_{k}(x)=f_{k-1}(f_{k}(x))$.

To train an autoencoder, the objective is to find the different weight vectors *W_i_* minimizing a specific objective function. We chose *binary crossentropy* as the objective function, which measures the error between the input *x* and the output *y*:

$$binary\;crossentropy(x,y)=\sum_{k=1}^{d}\left(x_{k}log(y_{k})+(1-x_{k})log(1-y_{k})\right)$$

We added two regularization penalty *α_w_* and *α_a_* for both weight vector *W_i_* and node activities *F*_1→_ *_k_* (*x*):

$$L(x,y)=binary\;crossentropy(x,y)+\sum_{i=1}^{d}(\alpha_{w}||W_{i}|{|}_{i}+\alpha_{a}||F_{1\to i}(x)|{|}_{2}^{2})$$

We set the three hidden layers in the autoencoder, which included 500, 100, and 500 nodes, respectively. The bottleneck layer of the autoencoder was adopted to generate novel characteristics from the four-level omics data. The penal values *α_w_* and *α_a_* were set as 0. 1 and 1 × 10^-7^, respectively. Finally, the autoencoder was trained by the gradient descent algorithm with 10 epochs and a batch size of 64.

### Selection of the Transformed Features and Sample Clustering

One hundred novel features were derived from the omics data based on the deep learning algorithm. For each of these transformed features, we performed the univariate Cox proportional-hazards regression analysis to find out the OS-related features (log-rank test, *P* < 0.05). Subsequently, we used these selected features to cluster the MIBC samples into groups based on the K-means clustering algorithm. The hazard ratio and the p-value derived from log-rank test were used to evaluate the prognostic differences.

### Genomic Analysis of TCGA Data

Somatic mutation data of TCGA BLCA and copy number segment data were downloaded from UCSC Xena database (https://xenabrowser.net/datapages/), respectively, and MIBC samples were extracted for downstream analysis. The mutation data was converted into “maf” format and visualized by Maftools (13). The segmentation file contains the segmented data for all the samples separated into S1 and S2 subgroups, and the recurrent frequency of each segment in each subgroup was calculated using GISTIC2 (14). The frequency of each chromosome cytoband in S1 and S2 was calculated smoothly from the files named “scores.gistic”, and then chi-square test was used to detect regions with significant differences in CNA frequency between S1 and S2 subtypes. Immune cell composition of MIBC was estimated from the expression data using the program CIBERSORT (15).

### Differential Expression Analysis and Functional Enrichment

Differentially expressed genes (DEGs) of TCGA data were detected by DESeq2 (16), and DEGs of microarray-based datasets were detected using the limma package (17) Hallmark gene set was downloaded from Molecular Signatures Database v7.0 (MSigDB, http://software.broadinstitute.org/gsea/msigdb/), and gene set enrichment analysis (GSEA) was performed using the R package “clusterProfiler” (18).

### Differential Methylation Analysis and Functional Enrichment

To test for differentially methylated CpG sites (DMS), we use the limma package. CpG site was defined as a DMS that |log2(fold-change)| of Beta value was more than 1 and adjusted p-value was less than 0.05. DMS located genes were extracted, and over-represent enrichment analysis was performed using the R package “clusterProfiler”.

### Data Partitioning and Prognostic Subgroup Robustness Assessment

All TCGA MIBC samples were randomly separated into training/testing datasets following a 4:1 split. Then, we build a supervised classification model using random forest, Naïve Bayes, k-Nearest Neighbor, and Adaboost algorithms. For the training dataset, we normalized each omics layer and calculated the p-value (Wilcox test) of each feature between these two prognostic subgroups. Then, we selected top features (50 for CNV, 100 for mRNA, 50 for miRNA, and 50 for CpG methylation) that are most correlated with subgroup labels based on the p-values. Then, we conducted 10-fold cross-validation with 10-time repeat to evaluate the predictive ability of the selected features.

During each repetition, different algorithms were applied (mentioned above), and receiver operating characteristic (ROC) curves were executed. The area under the curve (AUC) in all the repeats would provide us the predictive value of the classification. Once the AUC value was less than 0.7, the whole dataset would be re-split and the analysis would be re-started till the satisfying results were obtained. Finally, we select the best classification model with the highest AUC.

We selected the same features of each omics data in the testing dataset and predicted the label of each sample based on the classification model. The univariate Cox proportional-hazards regression analysis was performed to test the survival risk difference between the predicted groups.

For the out-group validation dataset, which only has a gene expression profile, we just use the overlapped features with the 100 mRNAs mentioned above to fit the classification model. The same tests were performed on TCGA testing dataset.

### Immunohistochemical (IHC) Staining and Assessment

Twenty-two MIBC samples were selected from Sun Yat-sen University Cancer Center, Guangzhou, China, between January 2015 and December 2015. Only samples with overall survival less than 1.5 years or over 5 years were taken into consideration in this study. IHC staining was performed using BenchMark ULTRA automatic immunostaining device according to the manufacturer’s instructions to analyze the KRT7 expression. In brief, the paraffin-embedded MIBC samples were sectioned and deparaffinized using EZ prep solution (BenchMark, Roche, Arizona, USA). The endogenous peroxidase activity was inhibited, and the sections were subjected to antigen retrieval in a cell-conditioning solution maintained at 95°C for 30 min. The sections with the primary antibody mouse anti-CK7 (MXB Biotechnologies Inc., Fuzhou, China, Kit-0021, 1:100 dilution) were incubated at 37°C for 1 h after adding Liquid crystal solution (BenchMark, Roche, Arizona, USA). A secondary antibody was then added at 37°C for 15 min, and signals were detected using the chromogen 3,3’-diaminobenzidine (DAB). The sections were counterstained with hematoxylin and then dehydrated and mounted on a coverslip. Staining proportion (0–100%) and staining strength (- to 4+) were measured for each sample, and an IHC score was calculated as follows:

$$S_{IHC}=S_{pro}+S_{str}$$

where *S_pro_* stands for the score of staining proportion (0%, *S_pro_* = 0; 1–20%, *S_pro_* = 1; 21–40%, *S_pro_* = 2; 41–60%, *S_pro_* = 3; 61–80%, *S_pro_* = 4; 81–100%, *S_pro_* = 5) and *S_str_* stands for the score of staining strength (-, *S_str_* = 0; +, *S_str_* = 1; ++, *S_str_* = 2; +++, *S_str_* = 3; ++++, *S_str_* = 4). The IHC score was used to measure the expression level of KRT7.

## Results

### The Identification of OS-Related Subtypes Based on TCGA Multi-Omics Data

**Figure 2:**
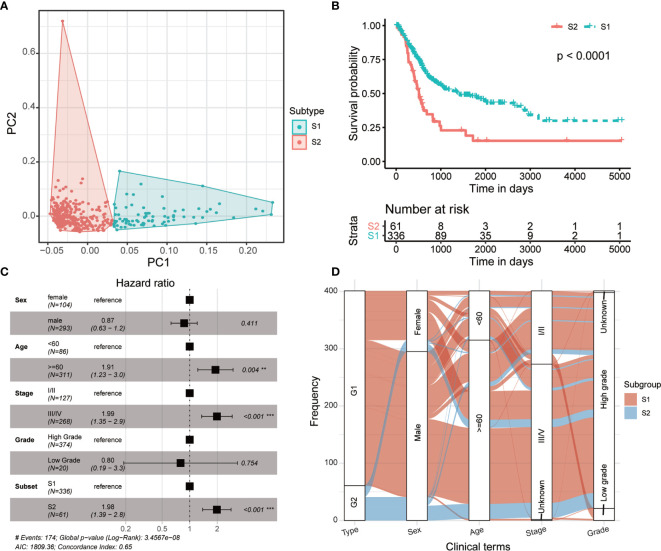
Two prognostic subtypes of MIBC were classified using multi-omics data-based deep learning framework. **(A)** Principal component analysis shows two distinguished MIBC subgroups clustered by K-means algorithm. **(B)** Kaplan-Meier curves show a significant difference of overall survival between MIBC subtypes. **(C)** Forest plot shows the multi-variates cox regression analysis result using general clinical characters as well as the predicted MIBC subtypes. **(D)** Distribution of the MIBC subtypes in various clinical phenotypes.

The multiple layers of genetic data were extracted from the TCGA database, and with the help of autoencoder-based deep learning algorithm, these data were stacked together (see *Materials and methods*). As a result, 100 new features were extracted from the bottleneck hidden layer, which represented the features of omics. We performed univariate Cox proportional-hazards regression analysis on these features and identified 98 features that were highly correlated with patients’ OS (*P* < 0.05, log-rank test; **Supplementary Table S1**). Subsequently, the MIBC patients were assigned into different clusters using K-means clustering algorithm referring to these OS-related features. We chose 2 as the optimal number of clusters (**Figure 2A**). Then, we conducted a univariate Cox proportional-hazards regression on the grouping result and observed that these two subtypes show a significant difference in OS outcomes (*P* = 8.11 × 10^-5^, log-rank test, **Figure 2B**). Furthermore, we performed multi-variates cox regression analysis using general clinical characters as well as the predicted subtypes, and the result shows that this molecular classification can be used as an independent prognostic indicator compared to general clinical information (**Figure 2C**). We further analyzed the relationship between the molecular subtyping and clinical information, and found that all patients from S2 were of high grade (**Figure 2D**).

### Molecular Differences Between These Two Prognostic Subtypes

**Figure 3:**
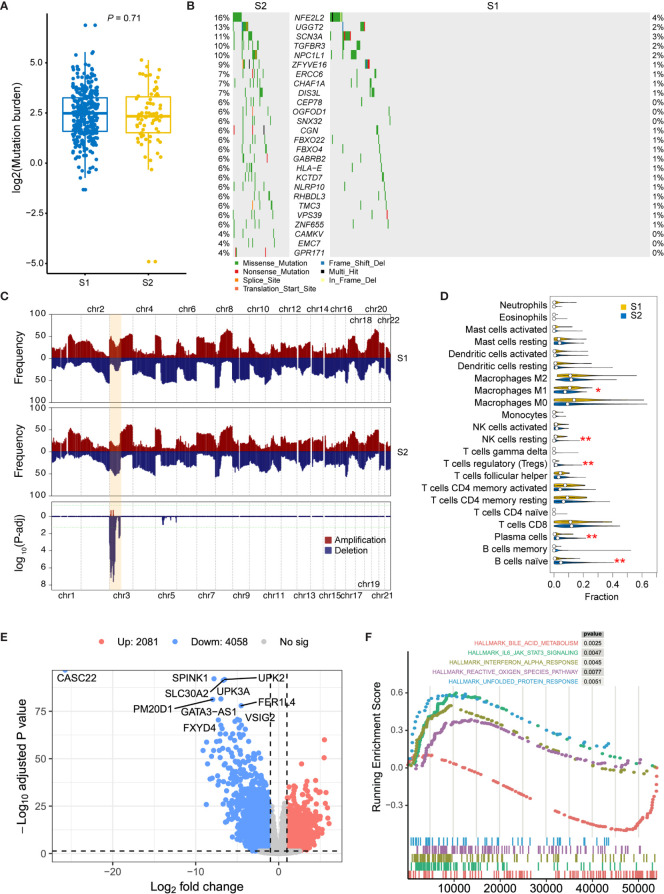
Molecular comparison between two prognostic MIBC subtypes. **(A)** Mutation burden of MIBCs for each tumor was compared in S1 and S2. **(B)** Oncoplot shows differentially mutated genes between two MIBC subtypes. Chi-square test is performed, and genes with *P* < 0.05 are displayed. **(C)** Frequency comparison between S1 and S2 of genome-wide copy number gain and loss. The CNV frequencies along genome of S1 and S2 are shown in top and middle pattern, respectively. All amplifications in MIBC cohort are shown in red, and all deletions are shown in blue. Chi-square test is performed for each cytoband, and the *P*-value distribution of each region was shown in the bottom module. Chromosome 3p, which contains all significant regions, is highlighted in orange. **(D)** Comparison of the immune cell compositions in S1 and S2. The immune cell contents were decomposed using CIBERSORT. Wilcox test is performed for each comparison, and significant entries are marked with asterisks (**P < 0.01; *P < 0.05). **(E)** Volcano plot shows the differentially expressed genes between high- and low-risk subtypes. Ten most significantly expressed genes are marked. **(F)** Top five hallmark gene sets from gene set enrichment between high- and low-risk subtypes.

In order to analyze the molecular characteristics of the two molecular subtypes, we firstly compared the differences in mutation and CNA levels between the two groups. There is no significant difference between the two subtypes in terms of mutation burden (**Figure 3A**). Several genes were found significantly mutated in S1, including *NFE2L2*, *UGGT2*, *SCN3A*, *TGFBR3*, and *NPC1L1* (**Figure 3B**). Besides, regions located on chromosome 3p have a significantly higher frequency of deletion in S2 patients (**Figure 3C** and **Supplementary Table 2**; adjusted *P*-value < 0.05, chi-square test), which contains some important tumor suppressor genes (TSGs) including *FANCD2*, *VHL*, *RPARG*, *XPC*, *TGFBR2*, *MLH1*, *SETD2*, and *RHOA*. Interestingly, TGF-Beta receptors were significantly altered in S2 at both SNV and CNV levels. Considering that transforming growth factor (TGF)-b is a key executor of immune homeostasis and tolerance, which can inhibit the expansion and function of many components of the immune system, we next performed immune decomposition for each sample and investigated the differences in immune components between the two molecular subtypes using CIBERSORT (15). As a result, tumors from S2 patients contained less M1 macrophages and resting NK cells, but more regulatory T cells, plasma cells, and naïve B cells (**Figure 3D**; *P* < 0.05, Wilcoxon signed-rank test).

Then, DEGs were derived by comparing the two prognostic subtypes, aiming to present the underlying mechanisms. A total of 6139 DEGs, including 2081 upregulated and 4058 downregulated genes, were detected with log_2_ fold change > 1 and FDR < 0.05 (**Figure 3E**). To investigate the functional difference between these two subtypes, we then performed Hallmark GSEA. In the top five most significantly enriched gene sets, we found that IL-6/JAK/STAT3 signaling, Interferon alpha response, reactive oxygen species, and unfolded protein response were activated in S2 subtype (high-risk group), while bile acid metabolism related genes were downregulated in this subtype (**Figure 3F** and **Supplementary Table 3**). Furthermore, we also performed differential methylation analysis between these two subtypes of MIBC. As a result, 40 hypermethylated CpG sites and 34 hypomethylated CpG sites were found in S2 group compared with S1 (**Supplementary Figure 1A**). The hypermethylated CpG site located genes had significantly enriched functions such as cell mitosis, cell junction, protein binding, endocytosis, AMPK signaling pathway, and VEGF signaling pathway (**Supplementary Figure 1B**), while the hypomethylated CpG sites were in genes related to GTPase binding and Ras guanyl-nucleotide exchange factor activity (**Supplementary Figure S1C**).

### Internal and External Validation of the Subtyping of MIBC

**Figure 4:**
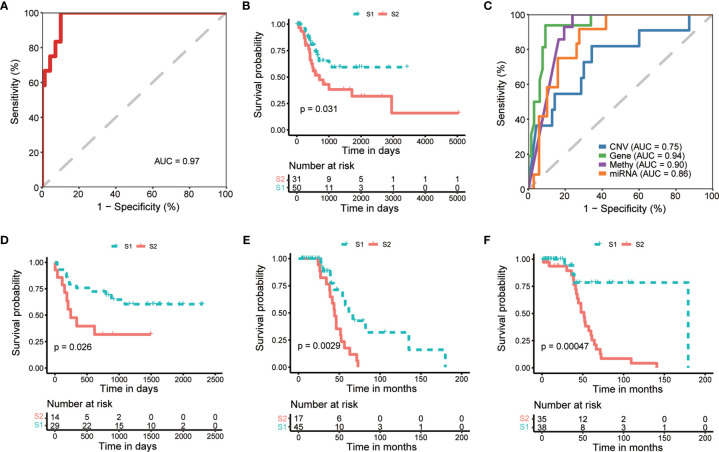
Internal and external validation of prognostic subtyping. **(A)** ROC analysis shows the robustness of subgroup classification in internal testing dataset using multi-omics data. **(B)** Kaplan-Meier curves show a significant difference of overall survival between subtypes predicted by multi-omics data in internal testing dataset. **(C)** ROC analysis shows the robustness of subgroup classification in internal testing dataset using each single omics data, respectively. **(D–F)**. Kaplan-Meier curves show a significant difference of overall survival between subtypes in external datasets, including GSE19915 **(D)** and two subsets of GSE48277 **(E, F)**, respectively.

To apply the identified classification into the prognosis of MIBC, we try to build a classification model of MIBC subtyping. We randomly selected 321 (80%) TCGA-MIBC cases as the training set and the other 81 (20%) MIBC cases as an internal validation set (**Table 1**). For the training set, we obtained the omics data at four levels (CNV profile, gene expression profile by RNA-seq, miRNA expression profile by miRNA-seq, and DNA methylation profile) and calculated the p-value for each feature from each omics data profile between the two subtypes by Wilcox test, respectively. The top features (50 for CNV, 100 for mRNA, 50 for miRNA, and 50 for CpG methylation) were selected for model training, which were mostly different between the two subgroups of MIBC. We perform 10-fold cross-validation with 10-time repeat to evaluate the predictive ability of the selected features. In each repeat, different algorithms were used separately to build supervised classification model, and the best model with highest AUC was selected for the internal validation (see *Materials and methods*). The same features were extracted from the internal validation cohort, and samples were classified into two different groups according to the prediction model. Considering the previous subtype labels of samples from internal validation set, we construct the ROC curve to evaluate the robustness of the supervised classification model (**Figure 4A**). The AUC value (AUC = 0.784) indicated the reliable robustness of the model. Kaplan–Meier survival curve showed that the classification model using cluster labels was robust to predict the survival-specific clusters (*P* = 0.031, log-rank test; **Figure 4B**).

**Table 1:** Basic information of training and validation datasets for MIBC subtyping model.

	Training set		Validation sets	
	TCGA	TCGA	GSE19915	GSE48277-1	GSE48277-2
**Total**	321	81	43	62	73
**Sex**					
Female	85 (26.5%)	21 (25.9%)	0 (0.0%)	13 (21.0%)	0 (0.0%)
Male	236 (73.5%)	60 (74.1%)	0 (0.0%)	49 (79.0%)	0 (0.0%)
N/A	0 (0.0%)	0 (0.0%)	43 (100.0%)	0 (0.0%)	73 (100.0%)
**Age**					
<60	72 (22.4%)	14 (17.3%)	0 (0%)	16 (25.8%)	13 (17.8%)
>=60	249 (77.6%)	67 (82.7%)	0 (0%)	46 (74.2%)	60 (82.2%)
N/A	0 (0.0%)	0 (0.0%)	43 (100.0%)	0 (0.0%)	0 (0.0%)
**Stage**					
II	106 (33.0%)	23 (28.4%)	19 (44.2%)	46 (74.2%)	42 (57.5%)
III	111 (34.6%)	27 (33.3%)	21 (48.8%)	15 (24.2%)	23 (31.5%)
IV	102 (31.8%)	31 (38.3%)	3 (7.0%)	1 (1.6%)	8 (11.0)
N/A	2 (0.6%)	0 (0.0%)	0 (0.0%)	0 (0.0%)	0 (0.0%)
**Grade**					
High	299 (93.1%)	79 (97.5%)	41 (95.3%)	0 (0.0%)	0 (0.0%)
Low	19 (5.9%)	2 (2.5%)	2 (4.7%)	0 (0.0%)	0 (0.0%)
N/A	3 (0.9%)	0 (0.0%)	0 (0.0%)	62 (100.0%)	73 (100.0%)

N/A, Not reported.

To expand the application of the prognostic subtyping, we also tested the stability of the identified classification using single-omics data from the internal validation dataset. We found the AUCs of gene expression data, miRNA expression profile, as well as methylation data were more than 0.8 (0.95, 0.90, and 0.87, respectively; **Figure 4C**), indicating the prediction robustness of these three single omics data. Then, we introduced three microarray-based gene expression datasets (GSE19915 and two subsets of GSE48277, **Table 1**) as external validation datasets to further validate our findings. Same expression features (the top 100 DEGs in training data) were extracted from each external validation datasets, and the supervised prediction model is tested in the same way of internal validation, respectively. The predicted two subtypes of MIBC also show significant OS differences in all the three cohorts (*P* = 0.026, *P* = 0.00094, and *P* = 0.00047, respectively, log-rank test; **Figures 4D–F**). This result indicates that this subtyping method could be effectively applied to classify MIBC patients into different risk levels.

### KRT7 Is a Marker Gene to Classify High-Risk and Low-Risk MIBC

**Figure 5:**
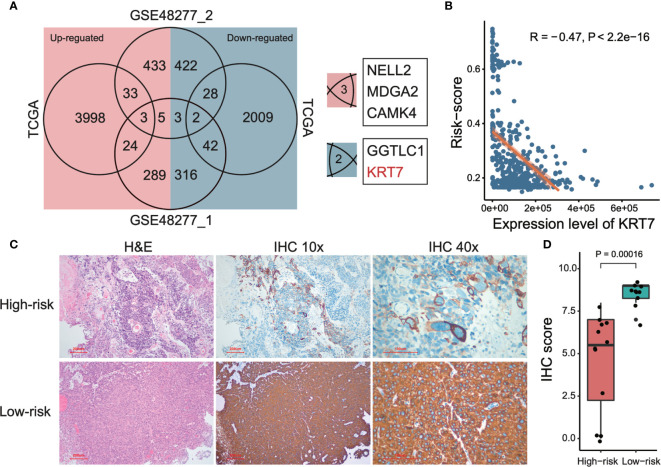
Detection and validation of risk-related markers of MIBCs. **(A)** Venn plot shows the overlaps of differentially expressed genes among TCGA and two subsets of GSE48277. **(B)** Correlation between tumor risk score and expression level of KRT7. TCGA data was used to perform this analysis. Both Pearson correlation coefficient (R) and P-value were calculated. **(C)** Representative images of KRT7 IHC staining in different risk types and corresponding H&E staining. **(D)** KRT7 protein expression was significantly decreased in high-risk MIBC specimens compared with low-risk MIBC tissues by IHC. For the details of calculating IHC score, please see *Materials and methods*.

In order to further investigate potential marker genes that distinguish high-risk and low-risk MIBCs, we integrated the DEGs between high-risk group and low-risk group of MIBC from datasets of TCGA and two subsets of GSE48277 (the expression matrix data of GSE19915 was centralized so that it is not considered in this analysis). As shown in **Figure 5A**, only three upregulated genes (*NELL2*, *MDGA2*, and *CAMK4*) and two downregulated genes (*GGTLC1* and *KRT7*) are overlapped among these three datasets, respectively. We selected *KRT7* (also named as CK7) as a candidate marker to distinguish high-risk and low-risk MIBC. As expected, the expression level of *KRT7* was negatively correlated with risk-score of MIBC (r = -0.47, *P* < 2.2 × 10^-16^; **Figure 5B**). We further verified this candidate at the protein level. Firstly, we examined the KRT7 expression in bladder tumors on the webserver of The Human Protein Atlas (https://www.proteinatlas.org/) and found that KRT7 protein was highly expressed in the low-grade bladder cancer cells but medially or lowly expressed in high-grade bladder cancer cells (**Supplementary Figure 2**). We next selected 22 MIBC samples and separated them into two distinct groups with different risks: the high-risk group (12 samples) were samples that OS < 1.5 years and samples from the low-risk group (10 samples) were survived over 5 years. As expected, KRT7 was significantly highly expressed in the low-risk MIBC (**Figures 5C, D** and **Supplementary Table 4**), which is further confirmed that KRT7 can be used as a marker to characterize MIBC risk.

## Discussion

Different levels of omics data could present diverse tumor landscape from different angles. It is required to integrate multi-omics data to describe the relations between clinical outcomes and molecular characteristics, then get a comprehensively understanding of cancer. In the present study, we construct an autoencoder-based deep learning framework to integrate CNV, gene expression, miRNA expression, as well as CpG methylation results to classify MIBC into two prognostic subtypes. The subtype S2 shows a significantly higher risk on overall survival and some specific genetic characters compared with the other subtype. We construct a robustness MIBC subtyping model depending on different omics layers and assessed the prognostic value in both internal and external validation datasets. We also detected KRT7 as a biomarker to reflect the risk of MIBC.

We found that in the poor prognosis group, chromosome 3p had a significantly higher frequency of deletions. Many tumor suppressor genes are located on chromosome 3p, including *TP53*, *VHL*, *MLH1*, *TGFBR2*, *THRB*, *RARB*, and *FHIT*. Loss of one copy of chromosome 3p is one of the most frequent and early events in human cancer, found in 96% of lung tumors and 78% of lung preneoplastic lesions (19). For cervical carcinoma (CC), researchers found that chromosome 3p deletions in precursor CIN lesions were smaller than the 3p losses found in the associated invasive CC (20). 3p arm loss has been associated with poorer prognosis for head and neck cancer as determined by reduced disease-free and overall survival of patients at early disease stage (21). These results suggest that the loss of chromosome 3p plays an important role in the occurrence and development of bladder cancer, and further analysis is needed. We detected 26 differentially mutated genes between S2 and S1. Some of these genes have been reported in previous tumor studies. For example, *NFE2L2* (the most significant gene that mutated in 16% of S2 but 4% in S1) has been reported in types of cancers. *NFE2L2* has long been considered a cytoprotective transcription factor, which is essential for the defense against oxidative stress, and activation of the *NFE2L2* pathway has been proposed as potential preventive strategy against carcinogenesis due to its function as a master regulator of the expression of antioxidant and detoxifying enzymes (22, 23). Reduced expression of *NFE2L2* are associated with poor outcome in breast cancer (24), ovarian cancer, and prostate cancer (25), but with favorable prognosis in cervical cancer (26), adrenocortical carcinoma, and kidney renal clear cell carcinoma (25), highlighting the dual role of *NFE2L2* in cancer. Remarkably, both mutation and CNA comparation show that TGF beta receptor was significantly altered in S2, indicating that the TGF-β signaling plays important roles in the prognostic impact in MIBC. One of the effects of this pathway is to enforce the immune homeostasis and tolerance, and disturbance of this pathway may influence the immune microenvironment of tumor. Interestingly, we found a variety of significant changes in immune cells between S1 and S2.

We investigate the gene expression and functional difference between the two prognostic subtypes. In the most significantly expressed genes shown in **Figure 3E**, lncRNA CASC22 has been reported that disrupting CASC22 was associated with a significantly increased risk of breast cancer (27). lncRNA FER1L4 also has been noticed as a favorable survival marker for endometrial carcinoma (28), colon cancer (29), and osteosarcoma (30). Interestingly, two UPK genes were significantly downregulated in high-risk MIBC subtype. UPK2 has been used as CTC markers of bladder cancer and got a satisfying result, which indicated a promising role for UPK2 mRNA detection using the circulatory blood of patients with urothelial cancer as a new staging marker (31). This is not consistent with our results. Besides, the most enriched gene sets were also demonstrated prognostic in previous studies. For example, elevated levels of IL-6 stimulate hyperactivation of JAK/STAT3 signaling, which is often associated with poor patient outcomes in colorectal cancer (32), breast cancer (33), oral cancer (34), and myeloma (35). Elevated levels of reactive oxygen species are also a common hallmark of cancer progression and resistance to treatment (36), and unfolded protein response was also demonstrated to play an important role in the establishment and progression of several cancers (37). To our surprise, we found a significant activation of interferon alpha (IFN-α) response. IFN-α is usually used as an adjuvant with bacillus Calmette-Guérin (BCG) in the non-invasive bladder cancer treatment. However, there is still a lack of evidence to demonstrate its benefit in preventing recurrences in intermediate-risk and high-risk patients (38). Although we only analyzed MIBC in this study, this result reminds us to be cautious of adjuvant IFN-a therapy, especially for the high-risk bladder tumors.

To demonstrate the robustness of the subtyping classification, we built the prediction models at single- and multi-omics level and tested them in internal and external validation cohorts. Both results show an effective distinction of OS between predicted groups. In association with clinical characteristics, we noticed that the DL-based subtyping presented more prognostic efficiency than other clinical indexes. Comparing with other previous genetic feature-based prognostic models, the DL-based subtyping method is more flexible that we can use the model based on single or multiple levels of genomics data. Moreover, the ROC curve shows that our method is more powerful than previous studies in single genomic level, for instance, mRNA expression level [AUC = 0.954 *vs*. AUC = 0.761 (39, 40)] and miRNA expression level [AUC = 0.901 *vs*. AUC = 0.663 (40)].

KRT7 is a member of the keratin gene family and is specifically expressed in the simple epithelia lining the cavities of the internal organs and in the gland ducts and blood vessels. KRT7 was reported as a predictive factor of various types of cancer, such as colorectal cancer (41) and renal clear cell carcinoma (42), but bad prognostic factor in esophageal squamous cell carcinoma (43) and pancreatic adenocarcinoma (44). KRT7 was also reported to promote epithelial-mesenchymal transition (EMT) of ovarian cancer (45). To the best of our knowledge, few studies reveal the relationship between KRT7 and MIBC. In this study, we report that KRT7 can be used as a biomarker that reflects the prognostic risk of MIBC. This conclusion comes from the analysis of both RNA and protein levels, highlighting the value of KRT7 in the clinical application of MIBC. However, the underlying biological mechanism still needs further research.

## Data Availability Statement

The original contributions presented in the study are included in the article/**Supplementary Material**. Further inquiries can be directed to the corresponding author.

## Author Contributions

XLZ, LS, and CW performed bioinformatics analysis. JL and XLZ performed IHC experiments. YH provided pathology support. XLZ and JW designed the research study. XLZ performed paper drafting. XPZ and XYZ performed paper editing. All authors contributed to the article and approved the submitted version.

## Funding

The work is supported by grants from National Natural Science Foundation of China (No. 81702791), Natural Science Basic Research Program of Shaanxi (2020JC-01), Medical Scientific Research Foundation of Guangdong Province, China (Grant No. A2019401), and National Key Research and Development program (No. 2017YFA0105900).

## Conflict of Interest

The authors declare that the research was conducted in the absence of any commercial or financial relationships that could be construed as a potential conflict of interest.

## Publisher’s Note

All claims expressed in this article are solely those of the authors and do not necessarily represent those of their affiliated organizations, or those of the publisher, the editors and the reviewers. Any product that may be evaluated in this article, or claim that may be made by its manufacturer, is not guaranteed or endorsed by the publisher.

## Supplementary Material

Supplementary Figure 1 Differential methylation analysis between S1 and S2. **(A)** Volcano plot shows differentially methylated CpG sites between S2 and S1. Sites with foldchange > 2 and adjusted *P*-value < 0.05 are considered to be significantly different. **(B)** Functional enrichment of hypermethylated CpG site related genes. Significantly enriched terms were defined as adjusted *P*-value < 0.05. Databases of GO, KEGG, Hallmark, and Reactome were included in this analysis, and top 10 most enriched terms of each database were shown in the figure. **(C)** Functional enrichment of hypomethylated CpG site related genes.

Supplementary Figure 2 Immunohistochemical results show the expression level of KRT7 in low-grade **(A)** and high-grade **(B)** MIBC patients. The IHC figures were selected and downloaded from the webserver of The Human Protein Atlas (https://www.proteinatlas.org/) after a specific query.
