# Supplementary figures and images for: Robust Prognostic Subtyping of Muscle-Invasive Bladder Cancer Revealed by Deep Learning-Based Multi-Omics Data Integration
Source: Front Oncol. 2021 Aug 6;11:689626. doi: 10.3389/fonc.2021.689626 (PMC8378227; doi:10.3389/fonc.2021.689626)

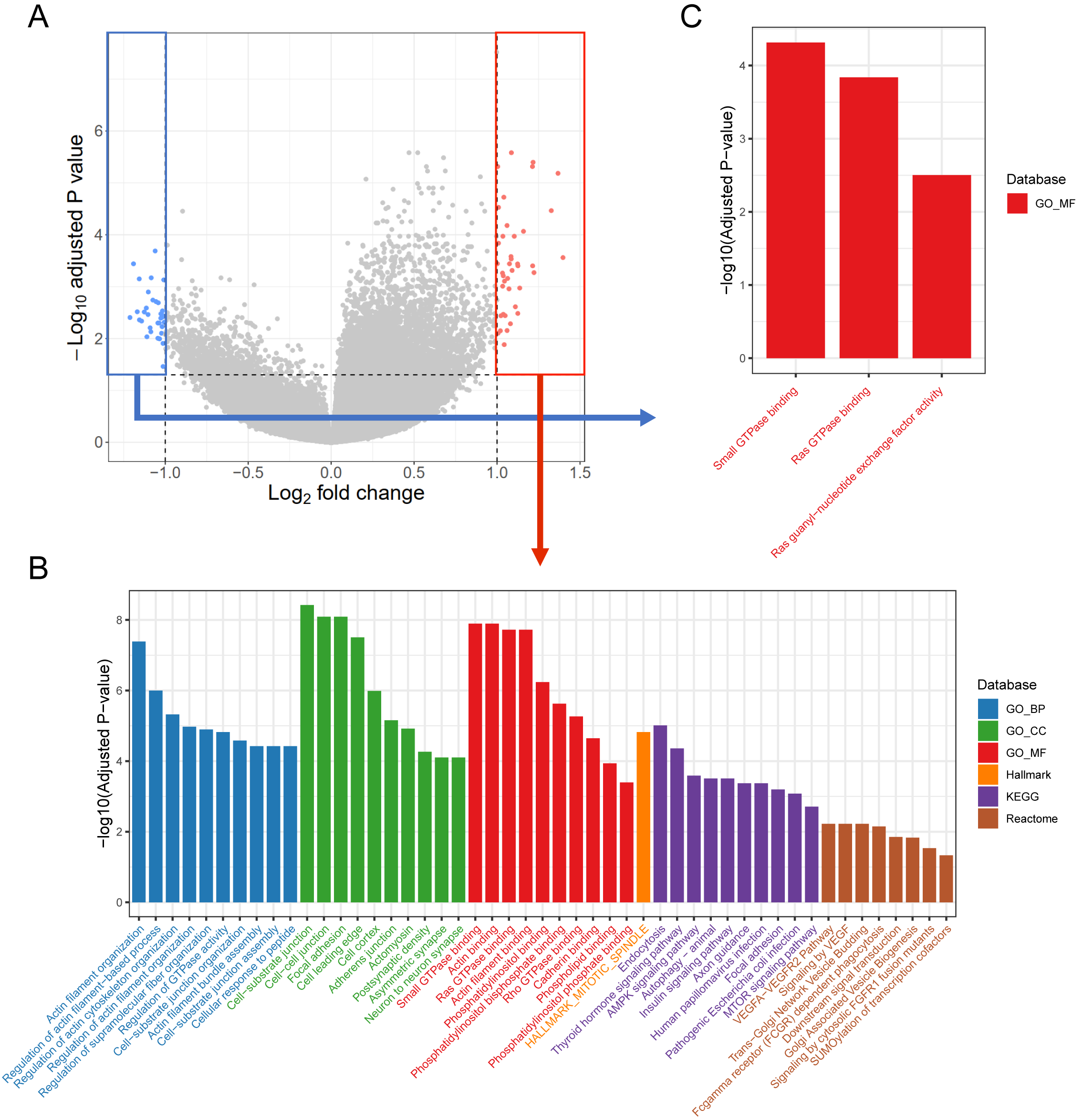

Supplement: Supplementary Figure 1 — Differential methylation analysis between S1 and S2. (A) Volcano plot shows differentially methylated CpG sites between S2 and S1. Sites with foldchange > 2 and adjusted P-value < 0.05 are considered to be significantly different. (B) Functional enrichment of hypermethylated CpG site related genes. Significantly enriched terms were defined as adjusted P-value < 0.05. Databases of GO, KEGG, Hallmark, and Reactome were included in this analysis, and top 10 most enriched terms of each database were shown in the figure. (C) Functional enrichment of hypomethylated CpG site related genes. [file Image_1.tif]

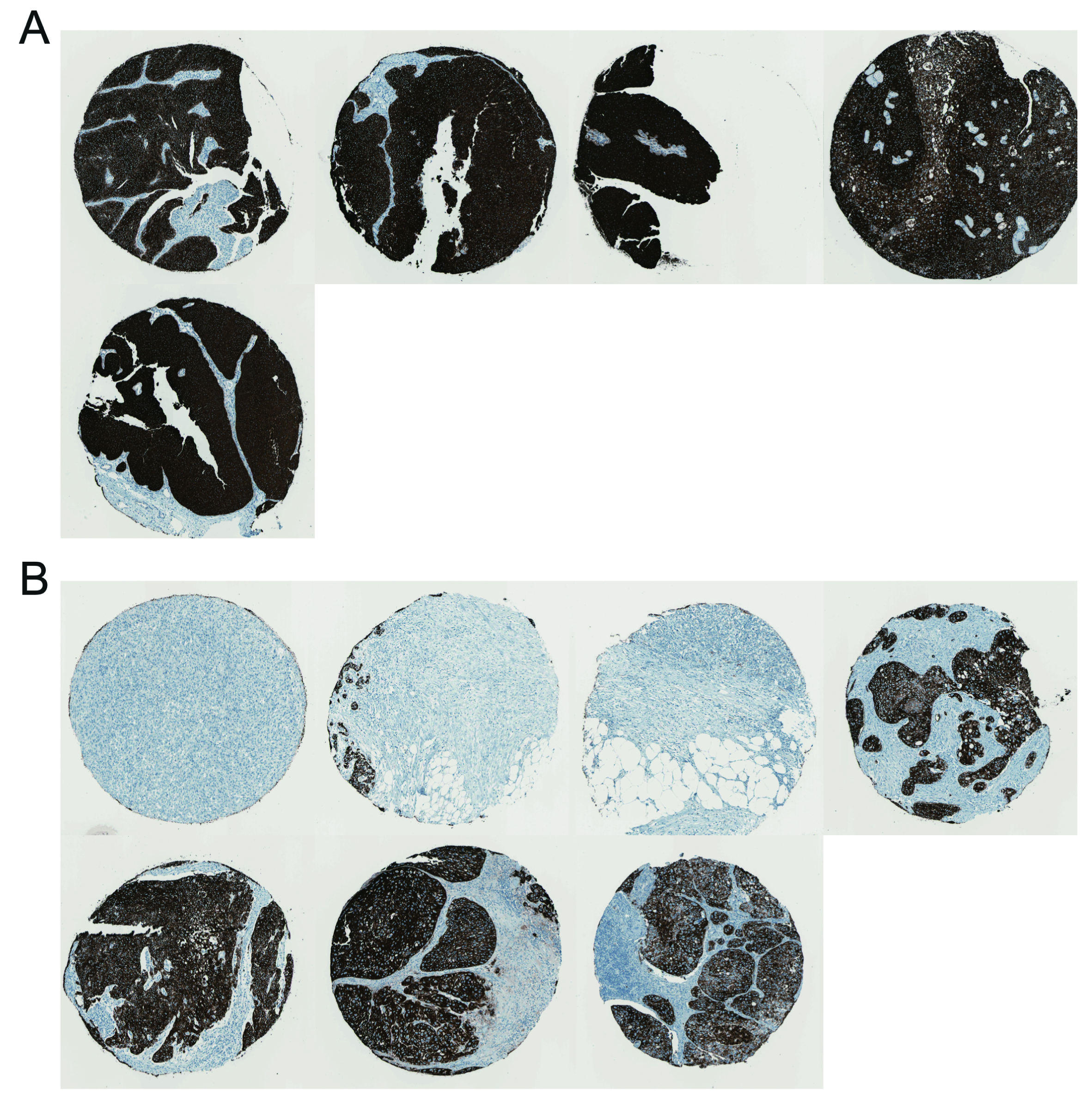

Supplement: Supplementary Figure 2 — Immunohistochemical results show the expression level of KRT7 in low-grade (A) and high-grade (B) MIBC patients. The IHC figures were selected and downloaded from the webserver of The Human Protein Atlas (https://www.proteinatlas.org/) after a specific query. [file Image_2.tif]
